# O fato materno: ansiedades epigenéticas e a ciência dos bebês biossociais

**DOI:** 10.1590/0102-311XPT085024

**Published:** 2024-08-19

**Authors:** João Paulo Gugliotti

**Affiliations:** 1 Faculdade de Medicina, Universidade de São Paulo, São Paulo, Brasil.

“*Você pode começar querendo destacar o papel do social e terminar com uma espécie de ideia biodeterminista de estratificação social*” ^1^.

Em seu novo livro, *The Maternal Imprint: The Contested Science of Maternal-Fetal Effects* [A Impressão Materna: A Ciência Contestada dos Efeitos Materno-fetais] ^2^, a historiadora e filósofa da ciência Sarah S. Richardson interroga os efeitos da gestação sobre a prole e o notável interesse científico acerca das implicações, a longo prazo, do ambiente intrauterino desde fins do século XIX. Richardson é catedrática de História da Ciência e de Estudos sobre Mulheres, Gênero e Sexualidade na Universidade de Harvard (Estados Unidos), onde coordena o laboratório Harvard GenderSci. Suas produções se concentram na interface entre ciência e os estudos de gênero, explorando questões relativas ao rigor conceitual e à responsabilidade social em pesquisas sobre sexo, gênero, sexualidade e reprodução.

A percepção social de que o ambiente intrauterino pode deixar uma marca permanente em seus descendentes suscitou debates e ansiedades terapêuticas da parte de leigos, mães, profissionais da saúde, cientistas, autoridades sanitárias, formuladores de políticas públicas, agências de pesquisa, empresas, além de movimentos por direitos sexuais e reprodutivos e agências internacionais de cooperação e desenvolvimento em saúde. Tais ansiedades não se dissiparam com o desenvolvimento de tecnologias avançadas e correntes teóricas advindas da biologia e medicina, tampouco em face da crescente especialização das disciplinas biossociais desde a descoberta do DNA em 1953. Ao contrário, se aprofundaram à medida que novos empreendimentos técnico-científicos produziram arrojados e controversos fatos sobre as interações entre o ambiente social, o DNA, a gravidez, o comportamento das mães e o futuro dos bebês.

Da era pré-DNA à pós-genômica, o social não foi, portanto, apagado ou rejeitado pelas produções mais influentes que se centram, contemporaneamente, na epigenética - que, grosso modo, é o estudo das mudanças moleculares fora do nosso DNA, que controlam como os genes se expressam. O “social”, por exemplo, é precisamente invocado por correntes a favor e contrárias ao pensamento genético prevalecente nos anos 1940-1950, constituindo dois quadros interpretativos sobre os efeitos materno-fetais: (i) a partir da contribuição do biólogo alemão August Weismann (1834-1914) e sua influente teoria do germoplasma, na qual postulou que não havia marca materna no desenvolvimento da prole, mas questões ecológicas como a nutrição, por exemplo; e (ii) pelos chamados “culturistas pré-natais” (*prenatal culturists*), cujas abordagens mesclavam saberes eugênicos e ideias progressistas sobre aprimoramento do corpo, desenvolvimento do cérebro e comportamento materno durante a gestação. Em síntese, e conforme a citação inicial ilustra, Richardson percorre habilmente o século XX até o presente e assegura que “o social” é o ponto de inflexão (e de disputa) sobre os efeitos materno-fetais em afirmações empíricas ou céticas desde Weismann, as quais criaram um ambiente pujante para experimentos que vão do peso dos bebês a efeitos teratogênicos a partir dos usos de substâncias tóxicas.

Nestas breves notas sobre o livro, e com base no argumento da autora, gostaria de refletir sobre aquilo que pode ser lido como “fato materno”, em ressonância ao chamado “fato social”. Da perspectiva sociológica, o termo me pareceu útil, pois, na sociologia durkheimiana, ele designa uma realidade externa, objetiva e que afeta a vida das pessoas na sociedade. Essa abordagem determinista encontrou terreno fértil nas Ciências Biológicas a partir dos anos 1950. Naquele contexto, o “social” passou a ser teorizado menos em razão de uma agenda científica comprometida em investigar e romper com desigualdades macroestruturais em saúde e os efeitos delas na gestação, mas mais como empreendimento científico e mercadológico, como um tipo de referente para explicações causais sobre doenças e o futuro bem-sucedido das gerações - agora, gene-centrado e fatorado pelo conceito de risco ^2^ ^,^ ^3^.

O “fato materno” é, portanto, a consubstanciação do “social” àqueles elementos ditos biológicos, provenientes da genética e dos estudos finais da eugenia que, apesar de rejeitá-lo, o incorporam de forma tautológica, biodeterminista e orientada ao elemento molecular, a fim de responsabilizar o corpo materno e torná-lo espaço de escrutínio, vigilância, controle e, não por acaso, culpa. Esse tropo da inclusão do “social” em correntes majoritariamente formadas por “culturistas pré-natais”, que ganham proeminência e certa hegemonia nos anos 1960 e adiante, retoma a velha questão da inclusão da variedade social na pesquisa científica - e das diferenças sociais com base em sexo, gênero, raça, etnia e idade - como um tipo de paradoxo ^3^. Richardson demonstra que nem todas as classificações de risco que buscam efeitos materno-fetais encontram os mesmos mecanismos de regulação e controle nos mesmos corpos. Nos anos 1950, quando o peso ao nascer começou a ser medido como uma variável demográfica, ele passou a significar uma medida de bem-estar biossocial do feto. A percepção científica do peso se alterava ao enquadrar mulheres brancas e afro-americanas, assim como as variáveis ecológicas relativas à dieta, uso de álcool, desempenho de atividades laborais intensas etc. O peso dos bebês se tornou, gradualmente, um importante biomarcador de sucesso ou fracasso da mãe e do pré-natal, sendo uma medida objetiva que viaja no tempo e no espaço.

“*Se você estiver estressada, você pode fazer ioga, ou ouvir música, porque cada movimento que você fizer você estará programando epigeneticamente a sua prole*” ^1^. Esse argumento parece convincente e, segundo Richardson, ele tem uma história. Se nos anos 1980 as preocupações com a gravidez e os efeitos materno-fetais despontaram no foco em toxinas, hoje o foco recai sobre as muitas formas de exposição a riscos. Com a epigenética, o comportamento materno ganha centralidade como eixo de controle, administração e medicalização, em que as mães são obrigadas, mesmo após o nascimento dos bebês, a refletir retrospectivamente sobre a causalidade dos problemas de seus filhos. O empenho de Richardson é, também, perceber como essas ideias são traduzidas em conselhos para mulheres, dados que informam políticas públicas e a prática clínica, em grande medida, de modo que essas recomendações podem fazer com que mulheres sintam que falharam antes mesmo de começar.

O livro pode ser lido de duas formas (ao menos): (i) como um livro historiográfico sobre as disputas e controvérsias científicas sobre o corpo feminino, a autonomia reprodutiva e as resistências permanentes à incorporação de uma perspectiva alinhada às demandas feministas e de reconhecimento das necessidades das mães como sujeitos de desejo, emoções e vontade; ou, ainda, (ii) como um exame sociológico, *queer* e antidisciplinar da noção de hereditariedade a partir de suas interações com discursos científicos influentes que têm se apoiado sobre inferências causais obscuras. O primeiro ponto é sintetizado por Richardson nos capítulos 1 a 4, principalmente; o segundo, nos capítulos 5 a 8.

Sobre o segundo ponto, mais especificamente, Richardson elabora considerações críticas sobre estudos afluentes da epigenética que buscaram, nas décadas de 2000 e 2010, sustentar suposições obscuras a respeito de teses ecológicas (comportamentais e ambientais), referentes às mães em período pré-natal e à metilação do DNA de seus filhos após grandes intervalos de tempo. Em alguns casos, tais estudos sugeriram relações de causalidade com desfechos como aumento ou diminuição do peso, disfunção metabólica e alteração do nível de citocinas. Contudo, esses métodos são enigmáticos, pois se concentram em hiatos temporais significativos (desde a gestação até a juventude e a fase adulta inicial), e cujas asserções de causalidade sobre efeitos originados da relação materna podem ser enormemente imprecisas.

À medida que os sujeitos se tornam mais longevos, novas especulações sobre os efeitos materno-fetais ganham capilaridade e importância no curso da vida, admitindo teorizações sobre sua intergeracionalidade. Isso posto, e embora esse não tenha sido o foco do livro, considero que as discussões críticas de Richardson sobre programação genética podem ser profícuas para o exame de estudos sobre o genoma e as doenças autoimunes e crônico-degenerativas na velhice, por exemplo. Se, por um lado, os efeitos materno-fetais têm sido explorados dentro de uma retórica biomédica que enxerga, no corpo materno, o ambiente de (in)sucesso da prole, por outro, é razoável inferir que tal diagnóstico não se limita apenas ao gênero, mas a determinações geracionais do processo saúde-doença, como é o caso de sujeitos em processo inicial e avançado de envelhecimento, e que são continuamente impelidos a consumir terapias e produtos de saúde com foco no envelhecimento ativo e bem-sucedido, com apelo ao bem-estar, qualidade de vida e satisfação.
